# Self-determination in secondary school students and their relationship with emotional intelligence and support for autonomy

**DOI:** 10.3389/fpsyg.2025.1571559

**Published:** 2025-07-09

**Authors:** Manuel Gómez-López, Daniel Mendoza-Castejón, Daniel Frías-López, David Manzano-Sánchez

**Affiliations:** ^1^Department of Physical Activity and Sport, Faculty of Sport Sciences, University of Murcia, Murcia, Spain; ^2^Department of Sports Sciences, Faculty of Medicine, Health and Sports, Universidad Europea de Madrid, Madrid, Spain; ^3^Department of Education, Faculty of Education, University of Almeria, Almería, Spain

**Keywords:** physical education, autonomy support, psychology, emotions, cluster profiles

## Abstract

**Introduction:**

In secondary education, a period characterized by multiple psychological, behavioral, and social changes in young people, academic motivation tends to decrease. This study focuses on physical education (PE) classes as a key space to positively influence students' motivation and emotional development, based on the Self-Determination Theory (SDT). This theory emphasizes the importance of satisfying three basic psychological needs (BPN): competence, autonomy, and social relationships, which in turn favor intrinsic motivation (IM) and emotional wellbeing.

**Methods:**

The study included 502 students (11-18 years old) and explored the relationship between motivational profiles high profile (19.7%), low profile (29.3%), and moderate profile (51%) and emotional intelligence (EI) and the perception of autonomy.

**Results:**

The results revealed that high self-determination profiles were associated with higher levels of IM, satisfaction of basic needs, and emotional wellbeing, while low self-determination profiles showed a motivation and negative consequences such as dissatisfaction and lower performance. In addition, it was observed that girls tended to present profiles of greater self-determination compared to boys (residual effect = 4.1), and that extracurricular sports practice was positively related to more self-determined motivational profiles (residual effect 7.7 and 7.5 in high and moderate self-determination profiles).

**Discussion:**

The findings highlight the need for adapted pedagogical strategies that promote autonomy, a positive climate and gender equity to maximize the positive impact of PE classes on the integral development of adolescents. In conclusion, PE classes can play a crucial role in improving student motivation and wellbeing if they are structured in a way that supports BPN and fosters an inclusive and motivating learning environment.

## 1 Introduction

The secondary education stage is a vital, dynamic period that significantly influences all facets of young people (Pfeifer and Berkman, 2018). Educators must address the numerous changes adolescents experience at psychological, behavioral, physical-physiological, and social levels (Shek et al., 2021). A major issue at this stage is student motivation in education, which tends to decline compared to earlier stages (Zheng et al., 2023). Motivation is a psychological mechanism that guides the direction, intensity, and persistence of behavior (Weinberg and Gould, 2007) toward academic goals (Alemany et al., 2015). One of the most prominent and widely used frameworks for studying motivation in both sports and educational contexts, particularly in physical education (PE), is the Self-Determination Theory (SDT) (Deci and Ryan, 1985, 1991, 2009; Ryan and Deci, 2000, 2017). This macro-theory examines the extent to which human behaviors are self-determined; that is, how voluntarily people engage in their actions.

The Basic Psychological Needs (BPN) model, a sub-theory of SDT, aims to explain human motivational processes across domains such as education by emphasizing the roles of competence, autonomy, and social relationships (Deci and Ryan, 1985; Ryan and Deci, 2017). In adolescence, the demand for satisfying these needs is particularly high (Vasconcellos et al., 2020), and when students perceive that they have control over their learning (autonomy), can achieve academic goals (competence), and feel connected to peers and teachers (relatedness), they are more likely to develop intrinsic motivation (IM), which is predictive of optimal academic development (Formento et al., 2023). Emotional factors such as hope and fear also influence the fulfillment of these needs, highlighting the importance of Emotional Intelligence (EI) and emotional regulation as complementary elements (Schüler et al., 2018). According to Salovey and Mayer's (1990) theory, fostering students' ability to identify, understand, use, and manage emotions enhances their adaptability to daily challenges. Research suggests a positive relationship between BPN satisfaction, self-determined motivation, and EI, while higher levels of external motivation (EM) or amotivation correlate negatively (Fernández-Espínola and Almagro, 2019). Although EI may not directly predict academic performance, it can influence it through mediating factors such as emotional wellbeing and effective learning strategies, which in turn support motivation (Nieto-Carracedo et al., 2024). Consequently, the interaction between SDT, BPN, and EI has been extensively examined in relation to the academic outcomes of secondary education students (Cachón-Zagalaz et al., 2023; MacCann et al., 2020); however, the present study aims to contribute new insights by incorporating potential differences based on self-determination profiles, daily physical activity practices, or students' gender when analyzed in combination.

Previous studies have separately analyzed factors influencing students' motivation and emotional development. One such factor is the teaching strategy, with those prioritizing student agency playing a significant role, regardless of gender or age (López-Lemus et al., 2023). Methodologies that foster autonomy—such as project-based learning and gamification—alongside a positive school climate that promotes healthy relationships and constructive competition, effectively enhance motivation and emotional outcomes (Ferriz-Valero et al., 2020; Zysberg and Schwabsky, 2020; Granero-Gallegos and Gómez López, 2020). Physical activity (PA), both curricular and extracurricular, is another key variable, linked to more self-determined motivation profiles, greater autonomy, and better emotional adaptability in adolescents (Vaquero-Solís et al., 2020; Barbosa et al., 2020). PE contributes to emotional intelligence (EI) development and fosters essential life skills such as self-discipline and resilience (Erickson et al., 2015; Fierro et al., 2019; Kayani et al., 2018). Intrinsic motivation (IM) in PE enhances engagement and performance, influenced by perceived motor competence and autonomy-supportive methodologies (Torres and Lizbeth, 2023; Carcamo-Oyarzun et al., 2023; Leo et al., 2020). Structured programs that promote social integration and cooperative problem-solving further support positive self-perception (Torres Pérez et al., 2024). Gender differences must be considered, as girls often report higher amotivation and external motivation, linked to lower autonomy and enjoyment (Romero-Parra et al., 2023; Huhtiniemi et al., 2019). Additionally, adolescent girls tend to show lower self-concept, self-competence, and self-esteem related to PA, with disparities widening due to shifting priorities, maturity differences, and varying interest in classroom activities (Fernández-Bustos et al., 2019; Alvariñas-Villaverde and Gonzólez-Valeiro, 2020).

For all these reasons, the present study aims to analyze the motivation of adolescents, their capacity for autonomy and the degree of EI they possess from their PE classes in order to guide new educational interventions. Thus, the secondary objectives of the study would be threefold: (a) To check the existing motivational profiles in PE students in Secondary Education according to their level of self-determination and the perception of satisfaction with the BPN; (b) To analyze the impact of the different motivational profiles on EI and the students' perception of autonomy; (c) To assess the differences in motivational profiles according to gender and the extracurricular sports practice of the students.

## 2 Methods

### 2.1 Design and participants

The study design was observational, descriptive and cross-sectional (Miller and Salkind, 2002). The study involved 502 secondary and baccalaureate students selected based on convenience and accessibility (265 boys, 52.8%; 236 girls, 47%) belonging to various schools in the Region of Murcia aged between 11 and 18 years (M = 14.163; SD = 1.68). Of the total number of students analyzed, 123 (24.5%) stated that they were not practitioners of extracurricular sports activities at the time of the study and 379 (75.5%) indicated that they were. The inclusion criteria were: participants must be between 11 and 18 years old; attend secondary schools; complete an informed consent form; and complete the questionnaires following established criteria to avoid exclusion.

### 2.2 Instruments

#### 2.2.1 Basic psychological needs (BPNES)

The version of the BPN in Exercise Scale, designed by Vlachopoulos and Michailidou (2006), validated in Spanish and adapted to PE by Moreno Murcia et al. (2008), was used. The questionnaire consists of 12 items grouped into three factors (four per dimension): autonomy (e.g., “I have the opportunity to choose how to perform the exercises”), competence (e.g., “I perform the exercises effectively”) and relationship with others (e.g., “I interact in a very friendly way with the rest of my classmates”). The answers are closed and respond to a Likert-type scale ranging from 1, a value that corresponds to totally disagree, to 5 that indicates that the students are in total agreement with what is proposed. The items are preceded by the phrase “In my Physical Education classes…”. In this study, the analysis of internal consistency has been satisfactory in the different subscales analyzed (autonomy, α = 0.757; competence, α = 0.732; relationship with others, α = 0.826).

#### 2.2.2 Support for autonomy

The Spanish version adapted to PE (LCQ-EF) by Granero-Gallegos et al. (2014) was used, which comes from the Learning Climate Questionnaire (LCQ; Williams and Deci, 1996), based on the Health-Care Climate Questionnaire (Williams et al., 1996). The questionnaire consists of 14 items to measure the support for autonomy by the teacher, through a dimension that is called: support for autonomy. In the instructions, the subjects are asked to indicate the degree of agreement with the items. The answers were collected on a 7-point polytomic item scale ranging from 1 (strongly disagree) to 2 (strongly agree). This scale showed a high internal consistency α = 0.963.

#### 2.2.3 Motivation

The Spanish adaptation (SMS-II-PE; Granero-Gallegos et al., 2018) of the Sport Motivation Scale-II by Pelletier et al. (2013). The instrument is composed of a total of 18 items to measure the individual level of motivation toward PE and distributed in six dimensions (3 items per dimension): intrinsic motivation (MI onwards) (e.g., “Because of the pleasure I feel while doing physical-sports activity”), extrinsic motivation (EM onwards) of integrated regulation (e.g., “Because the practice of physical-sports activity is a fundamental part of my life”), ME of identified regulation (e.g., “Because physical-sports activity is a way for me to develop”), introjected regulation ME (e.g., “Because I would feel bad if I did not participate and make an effort in classes”), external regulation ME (e.g., “Because I get reward from the people around me when I do it”), and amotivation (e.g., “I used to participate and work hard in classes, but now I wonder if I should continue to do so”). The scale used was preceded by the introductory sentence: “I participate and make an effort in Physical Education classes...”. The answers were collected on a Likert-type scale from 1 (strongly disagree) to 7 (strongly agree). In the present study, the analysis of internal consistency was: intrinsic motivation, α = 0.691; extrinsic motivation of integrated regulation, α = 0.732; extrinsic motivation of regulation identified, α = 0.714; extrinsic motivation of introjected regulation, α = 0.727; extrinsic motivation of extrinsic motivation, α = 0.739; amotivation, α = 0.717.

#### 2.2.4 Perceived emotional intelligence

The version adapted to Spanish (Fernández-Berrocal et al., 2004) from the original scale called the Trait Meta-Mood Scale (TMMS; Salovey et al., 1995). The scale measures the level of perceived EI through 24 items, distributed into three subscales of 8 items each, which evaluate emotional attention, clarity of feelings, and emotional repair. The emotional attention subscale expresses the degree to which people notice and think about their feelings (e.g., “I pay close attention to feelings”), the emotional clarity subscale assesses the ability to understand one's mood (e.g., “I am clear about my feelings”), the emotional repair subscale assesses the degree to which individuals moderate and regulate their feelings (e.g., “When I am sad, I think of all the pleasures of life”). The scale used was preceded by the introductory sentence: “Below you will find some affirmations about your emotions and feelings…” Responses were collected on a five-point Likert scale ranging from not at all agree (1) to strongly agree (5). In the present study, the analysis of internal consistency was: emotional attention, α = 0.867; clarity of feeling, α = 0.854; emotional repair, α = 0.818.

### 2.3 Procedure

In order to be able to attend the schools and carry out the fieldwork, authorization was obtained from the Management of each Center, School Council, and teachers of the PE subject of the assigned courses in the data collection, together with that of the parents of the students themselves through a letter explaining the objectives of the research study and how it would be carried out, accompanied by a copy of the instrument. Before beginning the fieldwork phase, the selected interviewers received specific training, following the guidelines outlined by Manzano et al. (1996). They were instructed in the techniques necessary to present the research project to students and faculty. Similarly, the interviewers reviewed each of the questionnaire questions, practiced in pairs, and discussed any questions or reactions the students might have on the day of the questionnaire administration. They were also trained in contact logging, the use of field sheets, and strategies to improve response rates and reduce potential bias. Finally, a pilot test was conducted with respondents similar to those who would later participate in the study, thereby allowing design flaws to be detected before the fieldwork began.

The students were informed of the purpose of the study, its voluntariness, absolute confidentiality of the answers and handling of the data, that there were no correct or incorrect answers and they were asked for the utmost sincerity and honesty when filling it out. The instrument was self-administered with massive application in a school day and with the consensus and prior training of the enumerators. All the fieldwork was carried out anonymously and always in the presence of both two interviewers and the professor of the PE subject. An average of ~20 min of class time was required to complete the questionnaire, varying slightly depending on the age of the students. Only those students who had the informed consent of their parents and/or guardians participated in the research. Finally, it should be noted that the positive report of the Research Ethics Commission of the University of Murcia (ID: 4447/2023) was previously obtained.

### 2.4 Data analysis

The first step was to perform a reliability analysis of all scales and then, the mahalanobis distance was used in order to detect and eliminate those atypical subjects or those that did not follow a logical pattern, also taking into account to perform an analysis of asymmetry and kurtosis, considered adequate to reduce less than two in asymmetry and seven in kurtosis (Curran et al., 1996). On the other hand, to perform the profile analysis, the variables were standardized in *Z*-scores. Finally, a sample of 502 subjects (522 initially) was obtained, and the reliability of the scales was again performed with the final sample, as well as an analysis of correlations between the various variables. Reliability was in any case higher than α = 0.60, a value considered adequate by Sturmey et al. (2005).

Secondly, a profile analysis was carried out, with the motivation scale acting with the variables of the self-determination index or DAI [intrinsic motivation ^*^ 2 + (identified regulation + introjected regulation)/2-(extrinsic regulation)-(amotivation ^*^ 2)] and the index of psychological mediators or IMP (autonomy + competence + social relationship)/3 as variables to perform them. To carry out the profiles, the suggestions of Hair et al. (1999) were followed, performing two steps after filtering the sample. Then, a hierarchical cluster analysis was performed using Ward's model and Euclidean distance squared, using *Z*-scores, including the motivation scale. The dendrogram suggested the elaboration of three or seven profiles. Next, the hypothesis of three profiles was confirmed with the k-means method, finding consistent results for this solution.

Thirdly, a multivariate analysis (MANOVA) was carried out taking into account the differences found in each of the variables under investigation. Additionally, the clusters were analyzed according to gender and the practice of sport outside the classroom, through an analysis of the chi-square value with 2 x 2 contingency tables, taking into account the residual effects. The statistical analysis was performed using the IBM SSPS 25.0 package.

## 3 Results

### 3.1 Descriptive and correlation analysis

The descriptive and correlation analysis can be seen in Table 1. As can be seen, the value of asymmetry and kurtosis was low for all variables, indicating an adequate homogeneity of these. On the other hand, it can be indicated that the most internal motivations were those that had a higher score, being in turn the need for social relations that also had a higher value. Analyzing the correlations, the results indicate that all variables were correlated with each other, except for amotivation with the need for competence, social relationship and satisfaction with life.

**Table 1:** Descriptive statistics and correlations between the subscales analyzed.

**Subscales**	***R***	***M***	***ST***	***A***	***K***	**α**	**1**	**2**	**3**	**4**	**5**	**6**	**7**	**8**	**9**	**10**	**11**	**12**	**13**	**14**
Autonomy	1–5	3.37	0.81	−0.350	−0.026	0.757	1	0.629^**^	0.465^**^	0.394^**^	0.312^**^	0.350^**^	0.409^**^	0.491^**^	0.459^**^	0.492^**^	0.465^**^	0.339^**^	−0.154^**^	0.559^**^
Competence	1–5	3.75	0.80	−0.433	−0.315	0.732		1	0.486^**^	0.514^**^	0.193^**^	0.370^**^	0.348^**^	0.480^**^	0.489^**^	0.535^**^	0.452^**^	0.285^**^	00.033	0.521^**^
Relationship with others	1–5	3.90	0.88	−0.785	0.281	0.826			1	0.509^**^	0.227^**^	0.391^**^	0.423^**^	0.354^**^	0.368^**^	0.397^**^	0.308^**^	0.174^**^	00.014	0.351^**^
Satisfaction with life	1–5	3.76	0.88	−0.629	−0.243	0.840				1	0.256^**^	0.527^**^	0.493^**^	0.389^**^	0.359^**^	0.383^**^	0.336^**^	0.211^**^	00.057	0.342^**^
Emotional_Attention	1–5	3.47	0.84	−0.318	−0.382	0.867					1	0.454^**^	0.415^**^	0.320^**^	0.323^**^	0.286^**^	0.324^**^	0.249^**^	0.165^**^	0.292^**^
Emotional_clarity	1–5	3.43	0.80	−0.314	−0.145	0.854						1	0.615^**^	0.308^**^	0.356^**^	0.314^**^	0.319^**^	0.202^**^	0.124^**^	0.305^**^
Emotional_repair	1–5	3.45	0.85	−0.344	−0.322	0.818							1	0.378^**^	0.409^**^	0.388^**^	0.334^**^	0.281^**^	0.157^**^	0.328^**^
Intrinsic_motivation	1–7	4.67	1.44	−0.309	−0.482	0.691								1	0.741^**^	0.758^**^	0.699^**^	0.406^**^	0.133^**^	0.497^**^
Integrated_regulation	1–7	4.48	1.50	−0.107	−0.750	−732									1	0.761^**^	0.673^**^	0.463^**^	0.174^**^	0.459^**^
Regulation_Identified	1–7	4.77	1.45	−0.281	−0.576	0.714										1	0.728^**^	0.396^**^	0.177^**^	0.503^**^
Regulation_introjected	1–7	4.66	1.42	−0.214	−0.558	0.727											1	0.406^**^	0.173^**^	0.458^**^
Regulation_extrinsic	1–7	3.81	1.62	0.103	−0.853	0.739												1	0.631^**^	0.304^**^
Amotivation	1–5	3.71	1.59	0.052	−0.793	0.717													1	0.116^**^
Autonomy support	1–5	4.75	1.53	−0.614	−0.363	0.963														1

** ρ < 0.01.

M, Media; ST, Standard deviation; A, Asymmetry; K, Kurtosis; α, Cronbach's alpha.

**Figure 1:**
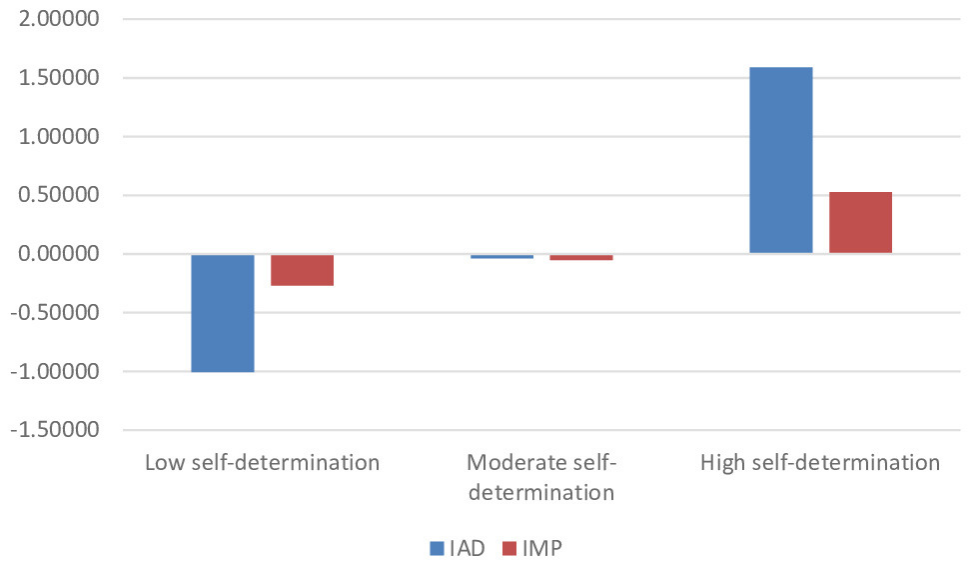
*Z*-score of cluster profiles.

Highlighting the two analyzed indexes and following the dendrogram, the results indicated the existence of three profiles that showed significant differences according to the self-determination index and the basic psychological mediators index, having a *Z*-score in the AMI and IMP of *Z* = −1.01 and −0.03 (low self-determination), *Z* = −0.27 and −0.05 (moderate self-determination) and *Z* = 1.6 and 0.53 (high self-determination. Visually it can be seen in Figure 1. The mean values for the ADI, a mean value of 2.32 (SD = 4.59) and for the IMP, a value of 3.67 (SD = 0.69), with asymmetry and kurtosis values of A = 0.66 and K = 0.48 (ADI) and A = −0.52 and K = 0.33 (IMP).

In the same way, Table 2 shows the three profiles and the values of the variables that contributed to the construction of the IMP and the ADI, showing a profile with high self-determination (high values of internal motivation and low values of extrinsic and amotivation), moderate self-determination (moderate values of internal and external motivation and amotivation) and low self-determination (high values of extrinsic regulation and amotivation, and moderate-low of internal).

**Table 2:** Mean values and standard deviation of the profiles analyzed.

	**Cluster profile**					
	**Low self-determination (*****n*** = **147; 29.3%)**		**High self-determination (*****n*** = **99; 19.7%)**		**Moderate self-determination (*****n*** = **256; 51%)**	
	**M**	**SD**	**M**	**SD**	**M**	**SD**
Intrinsic_motivation	3.84	1.42	5.95	0.93	4.66	1.25
Integrated regulation	4.01	1.49	5.57	1.28	4.33	1.39
Identified regulation	4.34	1.47	5.95	1.15	4.56	1.29
Introjected regulation	4.38	1.37	5.56	1.33	4.48	1.33
Extrinsic regulation	4.27	1.66	2.89	1.39	3.90	1.55
Amotivation	4.91	1.29	1.91	0.93	3.71	1.27
Autonomy	3.26	0.87	3.65	0.71	3.33	0.80
Competence	3.56	0.85	4.21	0.65	3.69	0.76
Relationship with others	3.66	1.00	4.26	0.71	3.90	0.83

### 3.2 Multivariate analysis according to motivational profiles

To test the interaction effects of the analyzed profiles and the variables of support for autonomy, life satisfaction, emotional attention, emotional clarity and emotional repair, a multivariate analysis of variance (MANOVA) was carried out in which the profile of the students acted as an independent variable and the subscales of the different variables, as dependent variables (Table 3). The results indicated that the homogeneity of the variance was evaluated by Box's *M*-test and the null hypothesis was rejected (Box's *M* = 4.202, *F* = 0.416 *p* = 0.045). That is why the value of Pillai's Trace was used instead of Wilks' Lamba following Field (2017). Multivariate contrast showed statistically significant differences in interaction effects according to profile (Pillai's Trace = 0.071 *F*_(3, 655)_, *p* = 0.001).

**Table 3:** Multivariate analysis of the variables under study according to profile.

	**Cluster profile**						***F***	***p***	***d***
	**Low self-determination**		**Moderate self-determination**		**High self-determination**				
	**M**	**ST**	**M**	**ST**	**M**	**ST**			
Autonomy support	4.40	1.60	4.72	1.48	5.32	1.41	50.102	0.000^**^	0.042
Satisfaction with life	3.56	0.97	3.73	0.83	4.11	0.80	17.991	0.000^**^	0.046
Emotional_Attention	3.37	0.90	3.47	0.80	3.63	0.85	3.982	0.061	0.011
Emotional_clarity	3.36	0.87	3.38	0.75	3.63	0.79	5.008	0.020^*^	0.016
Emotional_repair	3.37	0.92	3.41	0.79	3.67	0.85	6.398	0.011^*^	0.018

M, Mean; ST, Standard deviation.

** *p* < 0.001; ^*^p < 0.05.

Specifically, statistically significant differences were found in autonomy support, and life satisfaction (*p* < 0.001) and in emotional clarity and emotional reparation (*p* < 0.05). Performing the analysis differentiating according to the profiles, we found that, for autonomy support, the differences were between the profile with low self-determination and high self-determination (*p* < 0.001) and between high self-determination and moderate self-determination (*p* = 0.03). Regarding satisfaction with life, we found differences again between the most self-determined profile and the profile with low self-determination (*p* < 0.001) and moderate self-determination (*p* = 0.001). Emotional clarity was statistically different between the profile of high and low self-determination (*p* = 0.034) and moderate and high self-determination (*p* = 0.034). Finally, emotional repair differed for the profile with high self-determination compared to the profile with low self-determination (*p* = 0.016) and the profile with moderate self-determination (*p* = 0.022). Regarding emotional attention, no significant differences were found.

### 3.3 Differences in motivational profiles according to sex and extracurricular sports practice

To test the interaction effects of the sex variable and sports practice according to the cluster profiles, an analysis of contingency tables was carried out, checking the residual effects and using the chi-square test (Table 4).

**Table 4:** Analysis of categorical variables and the cluster profiles.

**Cluster profile**	**Value**	**Sex**		**Physical activity outside home**	
		**Male**	**Female**	**Yes**	**No**
Low self-determination	Count	81	66	103	44
	Residual	3.7	−3.7	−7.7	7.7
Moderate self-determination	Count	135	121	193	63
	Residual	0.4	−0.4	0.2	−0.2
High self-determination	Count	48	51	82	17
	Residual	−4.1	4.1	7.5	−7.5

Sex = Chi^2^ = 0.594; eTa = 0.015.

Physical activity outside home = Chi^2^ = 0.075; eTa = 0.102.

Analyzing Table 4, it can be seen that according to sex, the residual values were close to 4 in the case of the profile with low self-determination (with a value of 3.7 in favor of men) and that of high self-determination (with a value of 4.1 in women), taking into account a chi^2^ value of 0.075 not being significant. In the same way, looking at sports practice outside the home, if we find that those who practice sports outside the home predominate in the high self-determination profile with residual values of 7.5, unlike those who do not practice where the low self-determination profile has residual values of 7.7, taking into account a chi^2^ value of 0.594, not being significant. With this, it is concluded that there is a higher percentage of children and students who do not practice sport outside the home in the profile with low self-determination and the opposite with the high self-determination profile.

## 4 Discussion

The development of the discussion is organized according to the objectives of this study. The initial objective was “to check the existing motivational profiles in PE students in Secondary School according to their level of self-determination and satisfaction with the BPN.” As a second objective, it was intended to “analyze the impact of the different profiles on the EI and the perception of autonomy of students.” Finally, it sought to “assess the existing differences within the profiles according to sex and the performance of extracurricular sports practice.” The results obtained allow us to offer a comprehensive vision of the profiles of self-determination and their relationship with various relevant psychological variables in the educational context, thus allowing us to address the objectives set in a comprehensive way.

### 4.1 Motivational profiles in PE students

In relation to the first objective, the results of this study confirmed the existence of differentiated motivational profiles among PE students, supporting the central tenets of SDT (Ryan and Deci, 2017). Consistent with prior research, three distinct profiles emerged—highly self-determined, moderately self-determined, and low self-determined—mirroring those identified by García-González et al. (2019) and Fin et al. (2019), and reinforcing the notion that motivation in PE is not homogeneous.

Students in the high self-determination profile exhibited elevated levels of IM and integrated regulation, along with the highest satisfaction of basic psychological needs (BPN), especially social relatedness. This finding is in line with Wang et al. (2021), who highlighted how students with high self-determination demonstrate greater persistence due to the internalization of learning goals. Similarly, Vasconcellos et al. (2020) found that satisfaction of social relatedness was positively associated with IM and students' psychological wellbeing, while Aelterman et al. (2019) showed that support for autonomy boosts BPN satisfaction and promotes more self-determined motivation.

The correlation patterns between motivational variables (except for amotivation) further underscored the multidimensional nature of motivation, aligning with Vansteenkiste et al. (2018), who emphasized its continuum structure. Our findings also resemble those of Behzadnia et al. (2018), where satisfaction of BPN was positively associated with more self-determined forms of motivation and negatively with amotivation. In the present study, low self-determination was linked to high external regulation and amotivation and corresponded with lower BPN satisfaction—particularly autonomy and social relatedness—leading to less adaptive motivational outcomes. This pattern matches previous studies such as Rubio et al. (2020), who reported lower enjoyment and intention to be active among students with low self-determined profiles, and Cheon et al. (2018), who associated external regulation and amotivation with dissatisfaction of BPN and reduced engagement.

A particularly intriguing result was the emergence of a moderately self-determined profile, marked by intermediate levels of both autonomous and controlled motivation. This hybrid profile suggests a dynamic coexistence of motivational orientations, a complexity supported by Gillet et al. (2019), who argues that such profiles reflect the fluid and context-dependent nature of behavioral regulation. This notion is echoed in Fin et al. (2019), where a similar “moderate” profile was linked to contextual factors such as teacher support and classroom climate.

The relationship between BPN satisfaction and motivational profiles was consistent across studies. Our findings, like those of Trigueros et al. (2019), indicated that higher BPN satisfaction predicted more self-determined motivation. In addition, the perception of autonomy-supportive environments, as described by Haerens et al. (2015), was associated with stronger IM and identified regulation. Moreover, González-Cutre et al. (2019) emphasized the importance of novelty and variety in PE classes, which may also contribute to the formation of adaptive motivational profiles—an implication supported by our findings. Supporting this perspective, Sevil-Serrano et al. (2020) found that students with more self-determined motivational profiles reported greater enjoyment and less boredom in PE classes, underscoring the broader affective benefits of fostering autonomous motivation.

Interestingly, no statistically significant differences were observed in emotional attention between motivational profiles. This aligns with Petrides et al. (2016), who suggest that attention to emotions may function as a more stable trait, less influenced by variations in motivational orientation.

From an applied perspective, the identification of motivational profiles offers meaningful implications for teaching practice in PE. Tailoring pedagogical strategies to match the motivational needs of different student profiles could enhance engagement and learning outcomes. As Rodrigo-Ruíz et al. (2019) propose, promoting autonomy through the transfer of responsibilities may strengthen students' self-determined motivation. Similarly, fostering a positive social climate and offering meaningful choices could improve satisfaction of BPN, especially among students in low self-determination profiles, as suggested by Vasconcellos et al. (2020).

### 4.2 Emotional intelligence and the perception of autonomy

With respect to the second objective, our findings demonstrated that motivational profiles were significantly associated with students' EI and perception of autonomy. Students with a highly self-determined profile showed significantly greater emotional clarity and emotional repair, in contrast to those with moderate or low self-determination. These results align with previous studies linking self-determined motivation to emotional functioning (Gómez-López and Granero-Gallegos, 2020; Trigueros et al., 2019) and highlight how internal motivation may enhance adaptive emotional regulation skills. This suggests that fostering an environment that promotes the perception of autonomy and interpersonal relationships can increase not only self-determined motivation, but also students' emotional wellbeing.

This association can be interpreted through the lens of SDT (Ryan and Deci, 2017), which posits that the satisfaction of BPN—particularly autonomy and competence—facilitates both motivational quality and emotional development. Indeed, our findings resonate with those of Gómez-López and Granero-Gallegos (2020), who identified relatedness and competence as predictors of EI, especially in dimensions such as emotional clarity and repair. Similarly, Laborde et al. (2015) observed that these components of EI are more predictive of psychological wellbeing and performance than emotional attention.

Notably, emotional attention did not differ across motivational profiles, reinforcing the idea that this dimension may be less dependent on motivational orientation. This distinction has also been emphasized in studies by Arribas-Galarraga et al. (2017) and Salguero et al. (2012), who found that emotional clarity and regulation—not attention—are more strongly linked to adaptive functioning and reduced psychological distress. In line with this, Sánchez-Álvarez et al. (2016) suggested that high attention without clarity may even heighten vulnerability in controlling environments. Our findings thus contribute to the growing consensus that EI should be approached as a multidimensional construct, with differential links to motivation.

The relationship between self-determined motivation and broader indicators of wellbeing was also evident in students' reported life satisfaction. Those with highly self-determined profiles reported greater satisfaction, consistent with research by Fauzi et al. (2024), who found positive associations between IM, happiness, and life satisfaction in university contexts. These findings support the argument that motivation and emotional development are interdependent and context-sensitive.

Additionally, students who engaged in sports outside of school were more likely to belong to highly self-determined profiles and, in turn, displayed higher EI. This echoes findings by Trigueros et al. (2019), who reported that participation in extracurricular physical activity was linked to more autonomous motivation, suggesting that promoting active lifestyles may contribute to both motivational and emotional development.

Regarding autonomy perception, students with higher self-determination perceived greater support for autonomy in PE. This is consistent with research showing that autonomy-supportive environments foster more self-determined profiles (Vasconcellos et al., 2020; Escriva-Boulley et al., 2018). These environments promote volition and internalization, as noted by Ryan and Deci (2020), and have been associated with higher perceptions of autonomy across both secondary (Aelterman et al., 2019) and higher education (Gillet et al., 2019) contexts.

Importantly, this relationship appears to be bidirectional. On one hand, autonomy-supportive environments can promote self-determined motivation; on the other, students with autonomous profiles may be more sensitive to and appreciative of autonomy cues. This reciprocal dynamic, highlighted by Vansteenkiste et al. (2018), underscores the need for pedagogical strategies that consistently reinforce students' autonomy.

In practical terms, adopting autonomy-supportive teaching practices—such as providing meaningful choices, justifying learning tasks, and recognizing students' perspectives—can help cultivate more self-determined profiles and strengthen students' sense of autonomy (Reeve et al., 2019). By understanding how motivational profiles relate to emotional and perceptual outcomes, educators can better tailor interventions that enhance both psychological and educational experiences.

### 4.3 Differences by sex

In relation to the third objective, the analysis of motivational profiles based on students' sex and extracurricular physical activity revealed notable patterns. Contrary to traditional expectations, a higher proportion of girls appeared in the high self-determination profile, while boys were overrepresented in the low self-determination group. This finding contrasts with earlier research indicating higher IM among boys (Sevil et al., 2018; Lochbaum et al., 2016), often attributed to traditional gender roles that associate boys with competitiveness and physicality. While it should be noted that the residuals showed trends related to gender and sports practice, no significant differences were reflected, limiting the conclusions of the results.

However, recent studies point to an evolving picture. Monteiro et al. (2018), for example, found higher levels of IM among girls in PE contexts, aligning with the present findings and suggesting that motivational patterns may be shifting. This raises the possibility that observed differences are not fixed, but context-dependent—an idea supported by Vasconcellos et al. (2020), who emphasized that gender differences in PE motivation are shaped by cultural and educational factors.

This interpretation is further reinforced by the role of school-based interventions. Sánchez-Hernández et al. (2023) demonstrated that inclusive curricula, gender-sensitive teaching strategies, and efforts to dismantle stereotypes can promote self-determined motivation in girls. These contextual factors may explain the observed trend and highlight the relevance of educational policy and practice in shaping motivation.

Beyond the educational context, broader cultural transformations—such as increased visibility and support for female athletes and the progressive redefinition of gender roles—may also contribute to these motivational shifts. In particular, recent evidence suggests that the way female athletes are represented in the media can significantly influence their self-perception, identity, and participation in physical activity, thereby reshaping societal expectations and motivational outcomes (Sahin et al., 2024). As such, both micro-level influences (e.g., teaching approaches, school culture) and macro-level forces (e.g., media representation, social norms) interact to influence students' motivational profiles in PE.

Taken together, these findings suggest that gender-based motivational patterns in PE should not be viewed as static or universal. Instead, they appear to be malleable and responsive to both institutional interventions and societal change. This underscores the importance of adopting a multidimensional and context-sensitive lens when analyzing gender and motivation in PE, and calls for further research to explore these complex interactions across diverse educational environments.

### 4.4 Influence of extracurricular sports participation

Regarding extracurricular sports practice, the results revealed a clear trend: students who participated in extracurricular sports were more frequently found in the high self-determination motivational profile, while those who did not participate were overrepresented in the low self-determination profile. This pattern is consistent with a growing body of research showing that extracurricular physical activity is associated with more self-determined forms of motivation (Fin et al., 2019; Rubio et al., 2020; Leyton-Román et al., 2020). These studies consistently report higher levels of IM, identified regulation, and BPN satisfaction among students engaged in organized sports, reinforcing the role of extracurricular activity as a context that fosters internalization of motivation.

Monteiro et al. (2018) further emphasized that this motivation can transfer across contexts, with students showing greater self-determination in PE when they also engage in physical activity outside school. Vasconcellos et al. (2020) explained this transfer through the increased opportunities for autonomy, competence, and relatedness satisfaction offered by extracurricular sports. These opportunities seem to strengthen motivational quality, aligning with our finding of a strong residual association (7.5) between extracurricular sports and high self-determination.

However, it is important to interpret these patterns cautiously. Although the trends were clear, the chi-square analysis did not reach statistical significance, suggesting that other variables may mediate or moderate this relationship. As Standage and Vallerand (2014) proposed, factors such as the motivational climate or the degree of autonomy support provided by coaches could influence the strength of this association.

The relationship may also be bidirectional. Students with a more self-determined profile might be more inclined to seek out and maintain participation in extracurricular physical activity. This idea is supported by longitudinal data from Gunnell et al. (2020), who found that self-determined motivation predicted future physical activity engagement.

Conversely, students in the low self-determination profile were more likely to report not participating in extracurricular sports, as reflected in the residual value of 7.7. This group is of particular concern, as controlled motivation and amotivation have been associated with negative outcomes such as lower engagement, dropout, and future physical inactivity (Ntoumanis et al., 2021).

These findings highlight the importance of addressing potential barriers to extracurricular participation. As Fin et al. (2019) noted, factors such as socioeconomic status, family support, and perceived competence can limit access and motivation for engagement in physical activity outside of school. These contextual constraints may partially explain the motivational disparities observed among students.

Taken together, the evidence suggests that extracurricular physical activity can play a critical role in supporting more autonomous forms of motivation in PE, but the relationship is likely influenced by individual, social, and structural factors. Future research should continue to explore these dynamics using longitudinal and mixed-methods approaches to better understand causality and intervention strategies.

### 4.5 Limitations

Regarding the first objective, a relevant limitation is the cross-sectional nature of the research design, since, although three different motivational profiles were identified, this approach does not allow examining how these profiles can be modified over time or in response to different educational interventions. A longitudinal design would provide a much deeper understanding of the stability or variability of these motivational profiles. In addition, the study relied exclusively on self-report instruments to assess both motivation and BPN, which introduces potential methodological biases. Specifically, self-report measures are susceptible to social desirability bias, where participants may respond in a manner they perceive as more socially acceptable rather than reflecting their true thoughts or behaviors. This may compromise the validity of the data, particularly in sensitive constructs such as motivation and emotional perception. Moreover, self-report instruments depend heavily on the participants' level of introspection and self-awareness, which may be limited in adolescent populations. Incorporating complementary methods—such as teacher assessments, peer evaluations, or observational tools—could mitigate these limitations and provide a more accurate and multidimensional view of students' motivational profiles. Finally, the difference between students who participate in extracurricular sports and those who do not could influence the results.

In relation to the second objective, another significant limitation would be the lack of consideration of contextual variables that may influence the relationship between motivational profiles and outcome variables. Factors such as perceived motivational climate in the classroom, teacher teaching style, or PE curriculum characteristics were not examined, which could have provided a more nuanced understanding of how motivational profiles relate to EI and perception of autonomy. Another limitation is the absence of a more detailed analysis of the specific components of EI. Although differences in emotional clarity and emotional repair were found, the underlying reasons for these differences or how they might be related to specific aspects of motivational profiles were not explored in depth.

Finally, with respect to the third objective, an important limitation is the imbalance in the sample in terms of extracurricular sports practice, with 75.5% of practitioners compared to 24.5% who did not practice. This imbalance could have skewed the results and limited the ability to generalize conclusions to more diverse populations. In addition, the study did not delve into the specific characteristics of extracurricular sports practice, such as the type of sport practiced, frequency of practice, and intensity, which could influence motivational profiles. This additional information would have allowed for a more detailed analysis of how different forms of sports participation relate to motivation in PE classes. Finally, although some differences were found in relation to the students' sex and motivational profiles, the study did not explore in depth the underlying reasons for these differences or how they could be influenced by sociocultural factors. A more detailed consideration of gender norms and social expectations would have enriched the interpretation of these results.

### 4.6 Prospects

Based on the results found and the discussion presented in the study, the following future perspectives can be proposed for each of the three objectives:

Regarding the first objective, the findings on motivational profiles open several lines of future research: Longitudinal studies to examine how these profiles evolve over time, especially during the transition from primary to secondary education. Research on specific interventions designed to help students transition from less self-determined to more self-determined profiles. More detailed analysis of the contextual factors (e.g., the teacher's teaching style, classroom climate) that influence the formation of these motivational profiles.

In relation to the second objective, it is suggested: To investigate the causal relationship between motivational profiles and the development of EI through experimental studies. To explore how interventions focused on improving EI can influence students' motivation and perception of autonomy. To examine the mediating role of EI between BPN satisfaction and students' academic outcomes and wellbeing.

And lastly, based on the third objective, future research in this area could: Delve into the underlying reasons for differences in relation to sex in motivational profiles, considering sociocultural and psychological factors. Investigate how extracurricular sports experiences can be transferred to the PE context and vice versa. Develop and evaluate sex-specific intervention programs that promote more self-determined motivational profiles.

## 5 Conclusions

The findings of this study reinforce the relevance of promoting highly self-determined motivational profiles in PE classes. The identification of three distinct profiles—high, moderate, and low self-determination—offers a solid foundation for the development of tailored educational interventions aimed at enhancing student motivation. The results highlight the strong association between the satisfaction of BPN, self-determined motivation, and key psychological outcomes such as perceived autonomy support, life satisfaction, and EI. Moreover, the observed differences based on sex and engagement in extracurricular sports underscore the importance of implementing targeted strategies to address these disparities. These findings not only expand the current understanding of motivational processes in PE but also carry meaningful practical implications. Specifically, PE teachers can implement student-centered teaching methodologies that support BPN satisfaction. For example, using project-based learning and cooperative learning structures can foster autonomy and relatedness. Additionally, offering students meaningful choices within tasks, encouraging goal-setting and self-assessment, and providing informational rather than controlling feedback can enhance perceived competence and motivation. Finally, being mindful of students' diverse backgrounds and extracurricular involvement may help teachers adapt their instruction to be more inclusive and effective. Creating learning environments that incorporate these strategies may be key to improving students' motivation, emotional wellbeing, and ultimately their academic achievement in secondary PE classes.

## Data Availability

The raw data supporting the conclusions of this article will be made available by the authors, without undue reservation.

## References

[B1] Aelterman N. Vansteenkiste M. Haerens L. Soenens B. Fontaine J. R. Reeve J. (2019). Toward an integrative and fine-grained insight in motivating and demotivating teaching styles: the merits of a circumplex approach. J. Educ. Psychol. 111, 497–521. 10.1037/edu0000293

[B2] Alemany I. Campoy I. Ortiz M. Benzaquén R. (2015). Goal orientations in students of secondary: an analysis in a multicultural context. Publicaciones 45, 83–100.

[B3] Alvariñas-Villaverde M. Gonzólez-Valeiro M. (2020). Non-organised extracurricular physical and sport practice: gender, educational stage and physical activity index. Apunts. Educ. Fís. Deportes 141, 55–62. 10.5672/apunts.2014-0983.es.(2020/3).141.07

[B4] Arribas-Galarraga S. Saies E. Cecchini J. A. Arruza J. A. Luis-De-Cos I. (2017). The relationship between emotional intelligence, self-determined motivation and performance in canoeists. J. Hum. Sport Exerc. 12, 630–639. 10.14198/jhse.2017.123.07

[B5] Barbosa A. Whiting S. Simmonds P. Scotini Moreno R. Mendes R. Breda J. (2020). Physical activity and academic achievement: an umbrella review. Int. J. Environ. Res. Public Health 17:5972. 10.3390/ijerph17165972 32824593 PMC7460146

[B6] Behzadnia B. Adachi P. J. Deci E. L. Mohammadzadeh H. (2018). Associations between students' perceptions of physical education teachers' interpersonal styles and students' wellness, knowledge, performance, and intentions to persist at physical activity: a self-determination theory approach. Psychol. Sport Exerc. 39, 10–19. 10.1016/j.psychsport.2018.07.003

[B7] Cachón-Zagalaz J. Carrasco-Venturelli H. Sánchez-Zafra M. Zagalaz-Sánchez M. L. (2023). Motivation toward physical activity and healthy habits of adolescents: a systematic review. Children 10:659. 10.3390/children10040659 37189907 PMC10136410

[B8] Carcamo-Oyarzun J. Herrmann C. Gerlach E. Salvo-Garrido S. Estevan I. (2023). Motor competence, motivation and enjoyment in physical education to profile children in relation to physical activity behaviors. Phys. Educ. Sport Pedag. 10, 1–16. 10.1080/17408989.2023.2265399

[B9] Cheon S. H. Reeve J. Ntoumanis N. (2018). A needs-supportive intervention to help PE teachers enhance students' prosocial behavior and diminish antisocial behavior. Psychol. Sport Exerc. 44, 107–118. 10.1016/j.psychsport.2017.11.010

[B10] Curran P. West S. Finch F. (1996). The robustness of test statistics to nonnormality and specification error in confirmatory factor analysis. Psychol. Methods 1, 16–29. 10.1037/1082-989X.1.1.1626174714

[B11] Deci E. L. Ryan R. M. (1985). Intrinsic Motivation and Self-Determination in Human Behaviour. Champaign, IL: Plenum. 10.1007/978-1-4899-2271-7

[B12] Deci E. L. Ryan R. M. (1991). “A motivational approach to self: integration in personality,” in Nebraska Symposium on Motivation: Perspectives on Motivation, ed. R. Dienstbier (Lincoln, NE: University of Nebraska Press), 237–288. 2130258

[B13] Deci E. L. Ryan R. M. (2009). The “what” and “why” of goal pursuits: human needs and the self-determination of behavior. Psychol. Inq. 11, 227–268. 10.1207/S15327965PLI1104_01

[B14] Erickson K. C. Hillman C. Kramer A. (2015). Physical activity, brain, and cognition. Curr. Opin. Behav. Sci. 4, 27–32. 10.1016/j.cobeha.2015.01.005

[B15] Escriva-Boulley G. Tessier D. Ntoumanis N. Sarrazin P. (2018). Need-supportive professional development in elementary school physical education: effects of a cluster-randomized control trial on teachers' motivating style and student physical activity. Sport Exerc. Perform. Psychol. 7, 218–234. 10.1037/spy0000119

[B16] Fauzi M. M. W. Hussein N. Mohamed Razali M. Z. Anwar N. A. Omar N. (2024). Intrinsic motivation, life satisfaction and happiness: students at higher learning institution in Malaysia. Environ. Behav. Proc. J. 9, 137–143. 10.21834/e-bpj.v9iSI19.5767

[B17] Fernández-Berrocal P. Extremera N. Ramos N. (2004). Validity and reliability of the Spanish modified version of the trait meta-mood scale. Psychol. Rep. 94, 751–755 10.2466/pr0.94.3.751-755 15217021

[B18] Fernández-Bustos J. G. Infantes-Paniagua Á. Cuevas R. Contreras O. R. (2019). Effect of physical activity on self-concept: theoretical model on the mediation of body image and physical self-concept in adolescents. Front. Psychol. 10:1537. 10.3389/fpsyg.2019.01537 31354570 PMC6635469

[B19] Fernández-Espínola C. Almagro B. J. (2019). Relation between motivation and emotional intelligence in physical education: a systematic review. Retos 36, 584–589. 10.47197/retos.v36i36.64968

[B20] Ferriz-Valero A. Østerlie O. García Martínez S. García-Jaén M. (2020). Gamification in physical education: evaluation of impact on motivation and academic performance within higher education. Int. J. Environ. Res. Public Health 17:4465. 10.3390/ijerph17124465 32575919 PMC7344778

[B21] Field A. (2017). Discoring Statistics Using IBM SPSS Statistics, 5th Edn. London: Sage Publications.

[B22] Fierro S. Almagro B. J. Sáenz-López Buñuel P. (2019). Psychological needs, motivation and emotional intelligence in physical education. Rev. Electr. Interuniver. Formac. Prof. 22, 167–186. 10.6018/reifop.22.2.345241

[B23] Fin G. Moreno-Murcia J. A. León J. Baretta E. Nodari Júnior R. J. (2019). Interpersonal autonomy support style and its consequences in physical education classes. PLoS ONE 14:e0216609. 10.1371/journal.pone.0216609 31107894 PMC6527226

[B24] Formento A. C. Quílez-Robres A. Cortés-Pascual A. (2023). Motivation and academic performance in adolescence: a systematic meta-analytic review. Rev. Electr. Investig. Eval. Educ. 29, 1–23. 10.30827/relieve.v29i1.25110

[B25] García-González L. Sevil-Serrano J. Abós Á. Aelterman N. Haerens L. (2019). The role of task and ego-oriented climate in explaining students' bright and dark motivational experiences in physical education. Phys. Educ. Sport Pedag. 24, 344–358. 10.1080/17408989.2019.1592145

[B26] Gillet N. Morin A. J. Huyghebaert T. Burger L. Maillot A. Poulin A.. (2019). University students' need satisfaction trajectories: a growth mixture analysis. Learn. Instruct. 51, 54–65. 10.1016/j.learninstruc.2017.11.003

[B27] Gómez-López M. Granero-Gallegos A. (2020). Predicting emotional intelligence by meeting basic psychological needs in physical education classes. Int. J. Dev. Educ. Psychol. 1, 341–350. 10.17060/ijodaep.2020.n1.v1.1790

[B28] González-Cutre D. Romero-Elías M. Jiménez-Loaisa A. Beltrán-Carrillo V. J. Hagger M. S. (2019). Testing the need for novelty as a candidate need in basic psychological needs theory. Motiv. Emot. 44, 295–314. 10.1007/s11031-019-09812-7

[B29] Granero-Gallegos A. Baena-Extremera A. Pérez-Quero F. J. Ortiz-Camacho M. M. Bracho-Amador C. (2014). Spanish validation of the scale ≪intention to leisure-time in partake physical activity≫. Retos 26, 40–45. 10.47197/retos.v0i26.34392

[B30] Granero-Gallegos A. Gómez López M. (2020). Motivation and emotional intelligence in high school. Differences by gender. Int. J. Dev. Educ. Psychol. 1, 101–110. 10.17060/ijodaep.2020.n1.v1.1766

[B31] Granero-Gallegos A. Gómez-López M. González-Hernández J. Baena-Extremera A. Ortiz-Camacho M. M. (2018). Spanish adaptation and psychometric properties of the sport motivation scale-II with high school physical education students. Int. J. Environ. Res. Public Health 15:2768. 10.3390/ijerph15122768 30563291 PMC6313468

[B32] Gunnell K. E. Brunet J. Bélanger M. (2020). Linking psychological need satisfaction and physical activity to dimensions of health-related quality of life during adolescence: a test of direct, reciprocal, and mediating effects. J. Sport Exerc. Psychol. 42, 337–347. 10.1123/jsep.2015-0325 27736288

[B33] Haerens L. Aelterman N. Vansteenkiste M. Soenens B. Van Petegem S. (2015). Do perceived autonomy-supportive and controlling teaching relate to physical education students' motivational experiences through unique pathways? Distinguishing between the bright and dark side of motivation. Psychol. Sport Exerc. 16, 26–36. 10.1016/j.psychsport.2014.08.013

[B34] Hair J. F. Anderson R. E. Tatham R. L. Black W. C. (1999). Análisis Multivariante, 5th Edn. Madrid: Prentice Hall, IBERIA.

[B35] Huhtiniemi M. Sääkslahti A. Watt A. Jaakkola T. (2019). Associations among basic psychological needs, motivation and enjoyment within finnish physical education students. J. Sports Sci. Med. 18, 239–247. 31191093 PMC6544006

[B36] Kayani S. Kiyani T. Wang J. Zagalaz Sánchez M. L. Kayani S. Qurban H. (2018). Physical activity and academic performance: the mediating effect of self-esteem and depression. Sustainability 10:3633. 10.3390/su10103633

[B37] Laborde S. Dosseville F. Allen M. S. (2015). Emotional intelligence in sport and exercise: a systematic review. Scand. J. Med. Sci. Sports 26, 862–874. 10.1111/sms.12510 26104015

[B38] Leo F. M. Mouratidis A. Pulido J. J. López-Gajardo M. A. Sánchez-Oliva D. (2020). Perceived teachers' behavior and students' engagement in physical education: the mediating role of basic psychological needs and self-determined motivation. Phys. Educ. Sport Pedag. 27, 59–76. 10.1080/17408989.2020.1850667

[B39] Leyton-Román M. Núñez J. L. Jiménez-Castuera R. (2020). The importance of supporting student autonomy in physical education classes to improve intention to be physically active. Sustainability 13:3794. 10.3390/su12104251

[B40] Lochbaum M. Çetinkalp Z. K. Graham K. A. Wright T. Zazo R. (2016). Task and ego goal orientations in competitive sport: a quantitative review of the literature from 1989 to 2016. Kinesiol. Int. J. Fund. Appl. Kinesiol. 48, 3–29. 10.26582/k.48.1.14

[B41] López-Lemus I. Del-Villar Álvarez F. Gil-Arias A. Moreno-Domínguez A. (2023). Motivation and gender equity in physical education. Could hybridization of pedagogical models help us? Movimento 29:e29032. 10.22456/1982-8918.128080

[B42] MacCann C. Jiang Y. Brown L. Double K. S. Bucich M. Minbashian A. (2020). Emotional intelligence predicts academic performance: a meta-analysis. Psychol. Bull. 146, 150–186. 10.1037/bul0000219 31829667

[B43] Manzano V. G. Rojas A. J. Fernández J. S. (1996). Manual Para Encuestadores: Fundamentos de Trabajo de Campo. Ariel: Aspectos prácticos.

[B44] Miller D. C. Salkind N. J. (2002). “Elements of research design,” in: *Handbook of Research Design and Social Measurement*, eds. D. C. Miller and J. J. Salkind (Thousand Oaks, CA: SAGE Publications), 18. 10.4135/9781412984386.n9

[B45] Monteiro D. Teixeira D. S. Travassos B. Duarte-Mendes P. Moutão J. Machado S.. (2018). Perceived effort in football athletes: the role of achievement goal theory and self-determination theory. Front. Psychol. 9:1575. 10.3389/fpsyg.2018.01575 30210403 PMC6121108

[B46] Moreno Murcia J. C. González-Cutre Coll D. Chillón Garzón M. Parra Rojas N. (2008). Adaptation of the basic psychological needs in exercise scale to physical education. Rev. Mex. Psicolog. 25, 295–303.

[B47] Nieto-Carracedo A. Gómez-Iñiguez C. Tamayo L. A. Igartua J. (2024). Emotional intelligence and academic achievement relationship: emotional well-being, motivation, and learning strategies as mediating factors. Psicol. Educ. 30, 67–74. 10.5093/psed2024a739486118

[B48] Ntoumanis N. Ng J. Y. Y. Prestwich A. Quested E. Hancox J. E. Thøgersen-Ntoumani C.. (2021). A meta-analysis of self-determination theory-informed intervention studies in the health domain: effects on motivation, health behavior, physical, and psychological health. Health Psychol. Rev. 15, 214–244. 10.1080/17437199.2020.1718529 31983293

[B49] Pelletier L. G. Rocchi M. A. Vallerand R. J. Deci E. L. Ryan R. M. (2013). Validation of the revised sport motivation scale (SMS-II). Psychol. Sport Exerc. 14, 329–341. 10.1016/j.psychsport.2012.12.002

[B50] Petrides K. V. Mikolajczak M. Mavroveli S. Sanchez-Ruiz M. J. Furnham A. Pérez-González J. C. (2016). Developments in trait emotional intelligence research. Emot. Rev. 8, 335–341. 10.1177/1754073916650493

[B51] Pfeifer J. H. Berkman E. T. (2018). The development of self and identity in adolescence: neural evidence and implications for a value-based choice perspective on motivated behavior. Child Dev. Perspect. 12, 158–164. 10.1111/cdep.12279 31363361 PMC6667174

[B52] Reeve J. Cheon S. H. Jang H. (2019). “A teacher-focused intervention to enhance students' classroom engagement,” in Handbook of Student Engagement Interventions, eds. J. A. Fredricks, A. L. Reschly, and S. L. Christenson (Cambridge, MA: Academic Press), 87–102. 10.1016/B978-0-12-813413-9.00007-3

[B53] Rodrigo-Ruíz D. Cejudo J. Carlos Pérez-Gonzélez J. (2019). Compendium and analysis of measures of ability emotional intelligence. Rev. Iberoame. Diagn. Eval. Aval. Psicol. 2, 99–115.

[B54] Romero-Parra N. Solera-Alfonso A. Bores-García D. Delfa de la Morena J. M. (2023). Sex and educational level differences in physical activity and motivations to exercise among Spanish children and adolescents. Eur. J. Pediatr. 182, 533–542. 10.1007/s00431-022-04742-y 36482089

[B55] Rubio R. M. Granero-Gallegos A. Gómez-López M. (2020). The satisfaction of basic psychological needs in Physical Education classes and their relationship with satisfaction with life in adolescents. Rev. Complut. Educ. 31, 45–54. 10.5209/rced.61739

[B56] Ryan R. M. Deci E. L. (2000). Self determination theory and the facilitation of intrinsic motivation, social development, and well being. Am. Psychol. 55, 68–78. 10.1037/0003-066X.55.1.68 11392867

[B57] Ryan R. M. Deci E. L. (2017). Self-Determination Theory: Basic Psychological Needs in Motivation, Development, and Wellness. New York, NY: Guilford Publications. 10.1521/978.14625/28806

[B58] Ryan R. M. Deci E. L. (2020). Intrinsic and extrinsic motivation from a self-determination theory perspective: definitions, theory, practices, and future directions. Contemp. Educ. Psychol. 61:101860. 10.1016/j.cedpsych.2020.101860

[B59] Sahin D. Yildiz S. Demir M. (2024). The impact of media representation on female athlete identity and self-perception. Int. J. Sport Stud. Health 7, 1–9. 10.61838/kman.intjssh.7.3.1

[B60] Salguero J. M. Palomera R. Fernández-Berrocal P. (2012). Perceived emotional intelligence as predictor of psychological adjustment in adolescence: a 1-year prospective study. Eur. J. Psychol. Educ. 27, 21–34. 10.1007/s10212-011-0063-8

[B61] Salovey P. Mayer J. D. (1990). Emotional intelligence. Imagin. Cogn. Pers. 9, 185–211. 10.2190/DUGG-P24E-52WK-6CDG22612255

[B62] Salovey P. Mayer J. D. Goldman S. L. Turvey C. Palfai T. P. (1995). “Emotional attention, clarity, and repair:exploring emotional intelligence using the Trait Meta-Mood Scale,” in Emotion, ed. J. W. Pennebaker (Washington, DC: American Psychological Association), 125–151. 10.1037/10182-006

[B63] Sánchez-Álvarez N. Extremera N. Fernández-Berrocal P. (2016). The relation between emotional intelligence and subjective well-being: a meta-analytic investigation. J. Posit. Psychol. 11, 276–285. 10.1080/17439760.2015.105896830660750

[B64] Sánchez-Hernández N. Martos-García D. Soler S. Flintoff A. (2023). Quality education and gender equality as objectives of sustainable development in education: an experience with teachers in Spain. Sustainability 15:2487. 10.47197/retos.v48.9328727409075

[B65] Schüler J. Baumann N. Chasiotis A. Bender M. Baum I. (2018). Implicit motives and basic psychological needs. J. Pers. 87, 37–55. 10.1111/jopy.12431 30298518

[B66] Sevil J. Abós Á. Aibar A. Julián J. A. García-González L. (2018). Gender and corporal expression activity in physical education: effect of an intervention on students' motivational processes. Eur. Phys. Educ. Rev. 24, 372–389. 10.1177/1356336X15613463

[B67] Sevil-Serrano J. Aibar A. Abós Á. Generelo E. García-González L. (2020). Improving motivation for physical activity and physical education through a school-based intervention. J. Exp. Educ. 88, 363–383. 10.1080/00220973.2020.1764466

[B68] Shek D. T. Lin L. Ma C. M. S. Yu L. Leung J. T. Y. Wu F. K. Y.. (2021). Perceptions of adolescents, teachers and parents of life skills education and life skills in high school students in Hong Kong. Appl. Res. Qual. Life 16, 1847–1860. 10.1007/s11482-020-09848-9

[B69] Standage M. Vallerand R. J. (2014). “Motivation in sport and exercise groups,” in. *Group Dynamics in Exercise and Sport Psychology, 2nd Edn*., eds. J. Weinstein and M. Broda (London: Routledge), 567–589.

[B70] Sturmey P. Newton J. T. Cowley A. Bouras N. Holt G. (2005). The PAS–ADD checklist: independent replication of its psychometric properties in a community sample. Br. J. Psychiatr. 186, 319–323. 10.1192/bjp.186.4.319 15802689

[B71] Torres Pérez A. Gavala González J. Fernández García J. C. (2024). Physical activity practice and physical self-concept in students. Rev. EuroAm. Ciencias Deporte 13:26. 10.6018/sportk.541481

[B72] Torres G. Lizbeth D. (2023). La motivación intrínseca en las clases de educación física en estudiantes de bachillerato. Revisión sistemática. GADE Rev. Científ. 3, 145–159. 10.63549/rg.v3i2.218

[B73] Trigueros R. Aguilar-Parra J. M. Cangas A. J. Bermejo R. Ferrandiz C. López-Liria R. (2019). Influence of emotional intelligence, motivation and resilience on academic performance and the adoption of healthy lifestyle habits among adolescents. Int. J. Environ. Res. Public Health 16:2810. 10.3390/ijerph16162810 31394722 PMC6719049

[B74] Vansteenkiste M. Aelterman N. De Muynck G. J. Haerens L. Patall E. Reeve J. (2018). Fostering personal meaning and self-relevance: a self-determination theory perspective on internalization. J. Exp. Educ. 86, 30–49. 10.1080/00220973.2017.1381067

[B75] Vaquero-Solís M. Amado Alonso D. Sánchez-Oliva D. Sánchez-Miguel P. A. Iglesias-Gallego D. (2020). Emotional intelligence in adolescence: motivation and physical activity. Rev. Int. Med. Ciencias Act. Física Deporte 20, 119–131. 10.15366/rimcafd2020.77.008

[B76] Vasconcellos D. Parker P. D. Hilland T. Cinelli R. Owen K. B. Kapsal N.. (2020). Self-determination theory applied to physical education: a systematic review and meta-analysis. J. Educ. Psychol. 112, 1444–1469. 10.1037/edu000042031649571

[B77] Vlachopoulos S. P. Michailidou S. (2006). Development and initial validation of a measure of autonomy, competence, and relatedness in exercise: the basic psychological needs in exercise scale. Meas. Phys. Educ. Exerc. Sci. 10, 179–201. 10.1207/s15327841mpee1003_4

[B78] Wang C. K. J. Liu W. C. Kee Y. H. Chian L. K. (2021). Competence, autonomy, and relatedness in the classroom: understanding students' motivational processes using the self-determination theory. Heliyon 5:e01983. 10.1016/j.heliyon.2019.e01983 31372524 PMC6656925

[B79] Weinberg R. S. Gould D. (2007). Foundations of Sport and Exercise Psychology. Champaign, IL: Human Kinetics.

[B80] Williams G. C. Deci E. L. (1996). Internalization of biopsychosocial values by medical students: a test of self-determination theory. J. Pers. Soc. Psychol. 70, 767–779. 10.1037/0022-3514.70.4.767 8636897

[B81] Williams G. C. Grow V. M. Freedman Z. R. Ryan R. M. Deci E. L. (1996). Motivational predictors of weight loss and weight-loss maintenance. J. Pers. Soc. Psychol. 70, 115–126. 10.1037/0022-3514.70.1.115 8558405

[B82] Zheng W. Shen H. Belhaidas M. B. Zhao Y. Wang L. Yan J. (2023). The relationship between physical fitness and perceived well-being, motivation, and enjoyment in Chinese adolescents during physical education: a preliminary cross-sectional study. Children 10:111. 10.3390/children10010111 36670661 PMC9856568

[B83] Zysberg L. Schwabsky N. (2020). School climate, academic self-efficacy and student achievement. Educ. Psychol. 41, 467–482. 10.1080/01443410.2020.1813690

